# Association of Variants in *PLD1*, 3p24.1, and 10q11.21 Regions With Hirschsprung’s Disease in Han Chinese Population

**DOI:** 10.3389/fgene.2020.00738

**Published:** 2020-07-10

**Authors:** Wei-Bo Niu, Mei-Rong Bai, Huan-Lei Song, Yan-Jiao Lu, Wen-Jie Wu, Yi-Ming Gong, Xian-Xian Yu, Zhi-Liang Wei, Wen-Wen Yu, Bei-Lin Gu, Wei Cai, Xun Chu

**Affiliations:** ^1^Department of Pediatric Surgery, Xinhua Hospital, School of Medicine, Shanghai Jiao Tong University, Shanghai, China; ^2^Shanghai Key Laboratory of Pediatric Gastroenterology and Nutrition, Shanghai, China; ^3^Shanghai Institute of Pediatric Research, Shanghai, China

**Keywords:** association, Hirschsprung’s disease, case-control study, single nucleotide polymorphisms, Han Chinese population

## Abstract

**Background and Aims:** Hirschsprung’s disease (HSCR) is a rare genetically heterogeneous congenital disorder. A recent study based on whole genome sequencing demonstrated that common variants at four novel loci, which contained two intronic variants on *CASQ2* and *PLD1*, and intergenic variants located between *SLC4A7* and *EOMES* at 3p24.1, and between *LINC01518* and *LOC283028* at 10q11.21, were associated with HSCR susceptibility. To validate these associations with HSCR susceptibility, we performed a case–control study in a Han Chinese sample set.

**Methods:** We selected four previously identified single nucleotide polymorphisms (SNPs) for replication, along with tag SNPs to cover the four associated regions. In total, 61 SNPs were genotyped in 420 HSCR patients and 1,665 healthy controls from the Han Chinese population.

**Results:** None of the 14 tag SNPs in the *CASQ2* gene region, including the previously associated rs9428225, showed an association with HSCR. Among the 24 tag SNPs from the *SLC4A7*-*EOMES* region at 3p24.1, rs2642925 [odds ratio (OR) = 1.41, 95% confidence interval (95% CI) = 1.10–1.79; *P*_Additive_ = 0.007] and the previously associated SNP rs9851320 showed a suggestive association (OR = 1.22, 95% CI = 1.01–1.47; *P*_Additive_ = 0.042). A non-synonymous SNP, rs2287579, in *PLD1* showed a suggestive association with HSCR susceptibility (OR = 1.71, 95% CI = 1.18–2.46; *P*_Additive_ = 0.004). Additionally, the previously associated *PLD1* SNP rs12632766 showed a suggestive significance (OR = 1.20, 95% CI = 1.01–1.42, *P*_Additive_ = 0.038). In the *LINC01518*-*LOC283028* region at 10q11.21, three SNPs meet the study-wide significance threshold. Rs17153309 was the most associated SNP (OR = 1.60, 95% CI = 1.34–1.90; *P*_Additive_ = 1.13 × 10^–7^). The previously associated SNP rs1414027 also showed significant association (OR = 1.43, 95% CI = 1.20–1.70, *P*_Additive_ = 3.92 × 10^–5^). Two associated SNPs at 10q11.21 (rs1414027 and rs624804) were expression quantitative trait loci in digestive tract tissues from GTEx databases.

**Conclusions:** Our results confirmed that variants of the *LINC01518-LOC283028* region were associated with HSCR in the Han Chinese population. Additionally, the susceptibility of SNPs in the *LINC01518-LOC283028* region were associated with the expression levels of nearby genes. These results provide new insight into the pathogenesis of HSCR.

## Introduction

Hirschsprung’s Disease (HSCR) is a polygenic congenital disease (Wallace, 2011; Tilghman et al., 2019) characterized by the absence of enteric neurons in the variable length of the distal gastrointestinal tract (Heuckeroth, 2018). Children with HSCR manifest a functional intestinal obstruction. Constipation, chronic abdominal distension, vomiting, repeated enterocolitis, loss of appetite, and delayed growth and development caused by HSCR result in heavy burdens for individuals, families, and society (Amiel et al., 2008; Langer et al., 2017). The worldwide incidence rate of HSCR is 1/5,000 live births, and 2.8/10,000 live births in the Asian population (Amiel et al., 2008). Hirschsprung’s Disease patients can be divided into three subtypes according to the extent of aganglionosis: short-segment HSCR (S-HSCR), long-segment HSCR (L-HSCR), and total colonic aganglionosis (TCA; Tang et al., 2016).

It is well known that genetic factors play an essential role in the development of HSCR, with *RET* as the major risk gene, as genome-wide association studies (GWASs) using single nucleotide polymorphism (SNP) chip revealed that *RET*, *SEMA3*, and *NRG1* were associated with HSCR, which was also confirmed in subsequent independent studies (Garcia-Barcelo et al., 2009; Kim et al., 2014; Jiang et al., 2015; Fadista et al., 2018). Earlier studies adapting whole genome or exome sequencing strategies also revealed new risk variants with high penetrance. Using genome-wide copy number analysis and exome sequencing, *NRG3* was found to contribute to HSCR susceptibility (Tang et al., 2012; Yang et al., 2013). *FAT3* gene variants were seen to be significantly enriched in five HSCR families (Luzón-Toro et al., 2015). Whole exome sequencing coupled with functional analysis found that rare variants of *DENND3*, *NCLN*, *NUP98*, and *TBATA* were enriched in HSCR patients (Gui et al., 2017). Exome sequencing of samples from 190 patients of European ancestry revealed that seven genes, including *ACSS2*, *ADAMTS17*, *ENO3*, *FAM213A*, *SH3PXD2A*, *SLC27A4*, and *UBR4*, harbored rare HSCR risk variants with high penetrance (Tilghman et al., 2019). Notably, Tang et al. (2018) performed whole-genome sequence analyses of 464 patients with sporadic S-HSCR and 498 control individuals, and performed association analysis for both rare and common variants with HSCR susceptibility. Except for *BACE2* that harbors an excess of rare protein-altering variants, they found that a common variation in four novel loci was associated with HSCR, which contains two intronic variants on calsequestrin 2 (*CASQ2*) and phospholipase D1 (*PLD1*), and two intergenic regions (one between *SLC4A7* and *EOMES* on 3p24.1, another between *LINC01518* and *LOC283028* on 10q11.21).

We conducted a case–control study to further investigate the association of the common variations in *CASQ2*, intergenic region of *SLC4A7*-*EOMES*, *PLD1*, and intergenic region of *LINC01518*-*LOC283028* with HSCR susceptibility. We selected previously identified SNPs from the study of Tang et al. (2018) and tag SNPs of the four associated regions. In total, 61 SNPs were genotyped in 420 patients with HSCR and 1,665 healthy controls within the Han Chinese population.

## Materials and Methods

### Subjects

Study design and protocol conformed to the ethical guidelines of the Declaration of Helsinki and were approved by the Ethics Committee of the Xin Hua Hospital affiliated to Shanghai Jiao Tong University School of Medicine. Each individual, or the legal guardians of each child, received a detailed description of the purpose of this study and signed a written informed consent form. Sporadic HSCR patients were recruited from people who had received treatments in Xinhua hospital, affiliated to Shanghai Jiao Tong University school of Medicine, between 2008 and 2018. Diagnosis of HSCR was determined by histological examination of biopsy specimens for the absence of the enteric ganglia. We recruited 420 sporadic patients (322 males and 98 females, the male: female ratio of 3.29:1) with HSCR (323/58/39 for S-HSCR/L-HSCR/TCA), and the mean age of HSCR patients was 1.16 ± 1.71 years. A total of 1,665 gender-matched healthy controls, who visited Xinhua hospital for routine health check-ups, were randomly selected as controls, including 1281 males and 384 females (the male: female ratio of 3.34:1) with a mean age of 36.14 ± 7.54 years. Each control subject was in good health and without a history of HSCR or any other neurological disorders. All the cases and controls were unrelated individuals of Han Chinese origin. Genomic DNA was extracted from peripheral blood leukocytes using the QIAamp DNA Blood Mini Kit, according to the manufacturer’s protocol (Qiagen, Hilden, Germany).

### SNP Selection

Four new loci were identified in a previous study by Tang et al. (2018) showing a moderate association (*P* < 1 × 10^–6^) with HSCR, which includes 2 intergenic (rs1414027 between *LINC01518* and *LOC283028* on 10q11.21, and rs9851320 between *SLC4A7* and *EOMES* on 3p24.1) and 2 intronic variants (rs12632766 on *PLD1* and rs9428225 on *CASQ2*) (Tang et al., 2018). We genotyped these four SNPs for replication and selected tag SNPs to cover the common variation of these associated regions. The Genome Variation Server^1^ with MAF (minor allele frequency) ≥ 0.01 and *r*^2^ ≥ 0.8 based on the HapMap HCB (Han Chinese in Beijing) data was used to select. The four reported SNPs were forced to be tag SNPs in the tag SNP selection procedure. We selected 14 tag SNPs, including rs9428225 to cover the *CASQ2* gene region and a 10 kb region flanking the 5′ and 3′ end of the gene. Twenty-four tag SNPs, including rs9851320, were selected to cover the intergenic region between *SLC4A7* and *EOMES* on 3p24.1. Additionally, we selected 13 tag SNPs, including rs12632766, to cover the *PLD1* gene region and a 10 kb region flanking the 5′ and 3′ end of the gene. Ten SNPs, including rs1414027, were selected to cover the intergenic region between *LINC01518* and *LOC283028.* In total we genotyped 61 SNPs to investigate the associations of the four regions with HSCR susceptibility.

### Genotyping

Sixty-one SNPs were genotyped using a Fluidigm 192.24 Dynamic Array with EP1 (Fluidigm Corp., CA). Allele-specific fluorescent (FAM or VIC) primers and common reverse primers were employed for genotyping. We used EP1 SNP Genotyping Analysis software to analyze the data. We placed one duplicate sample on each 96-well sample plate to assess genotyping accuracy. The PCR was conducted on a total volume of 4 μl per inlet. The primer sequences and PCR reaction conditions are shown in Supplementary Tables S1,S2.

### *In silico* Functional Annotation

Functional annotation of associated SNPs was explored by HaploReg v4.1. The expression quantitative trait locus (eQTL) evidence of disease-associated SNPs was investigated using GTEx database Release V8 (dbGap Accession phs000424.v8.p2) (GTExConsortium, 2015).

To quantify the probability that the HSCR association and eQTL signals at a given locus were attributable to a shared causal variant, we conducted colocalization analysis by coloc using our association summary statistics and eQTLs data from GTEx (Guo et al., 2015). eQTLs originating from all digestive tract regions were extracted from the GTEx database, including tissues of esophagus muscularis (*n* = 465), esophagus mucosa (*n* = 497), esophagus – gastroesophageal junction (*n* = 330), stomach (*n* = 324), small intestine – terminal ileum (*n* = 174), colon transverse (*n* = 368), and colon – sigmoid (*n* = 318). We restricted analyses to genes within 1 Mb of the associated SNPs of interest [eQTL *P* < 0.01 and *m*-value (Posterior Probability from METASOFT) > 0.9] and ran coloc.abf with default priors. We considered coloc tests with a Posterior probability of hypothesis 4 (PPH4) ≥ 0.9 or PPH4.ABF > 0.5 as having strong or moderate evidence for colocalization.

### Statistical Analysis

Hardy–Weinberg equilibrium tests and quality control were performed using PLINK1.9 (Purcell et al., 2007). Linkage disequilibrium of all pairs of SNPs was estimated using D’ and *r*^2^ according to the standard measurement. Haplotype block was determined using Haploview 4.2 (Barrett et al., 2005). Association analysis was performed using PLINK1.9 under the additive genetic model. SNP–SNP interaction was assessed using a logistic regression approach with additive encoding as implemented in PLINK1.9 (Purcell et al., 2007). Conditional logistic analysis was performed to find SNPs with an independent effect by adding the top associated SNPs as covariates in the model. We computed statistical power using the Genetic Power Calculator^2^ (Purcell et al., 2003). A study-wide level *P* value < 0.00086 (0.05/58) was considered statistically significant.

## Results

### Case-Control Association Study

**FIGURE 1:**
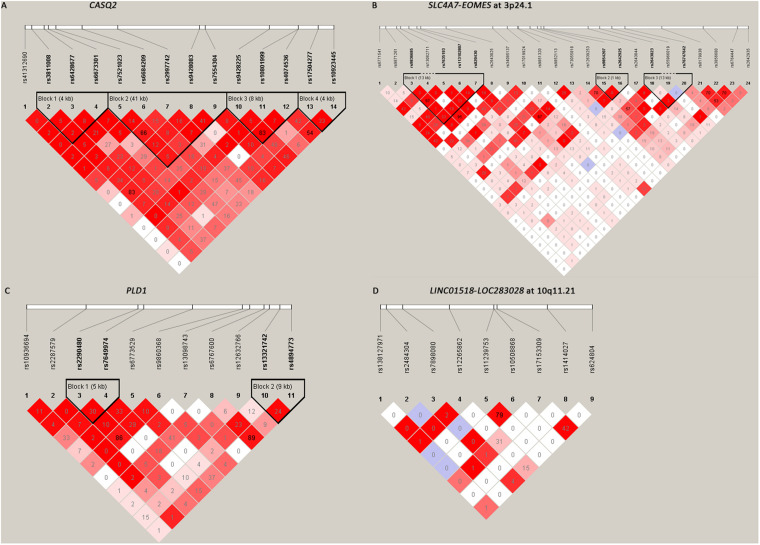
The linkage disequilibrium (LD) patterns of the four loci. **(A)** 14 SNPs in *CASQ2* regions; **(B)** 24 SNPs between *SLC4A7* and *EOMES* at 3p24.1; **(C)** 11 SNPs in the *PLD1* region; **(D)** 9 SNPs between *LINC01518* and *LOC283028* at 10q11.21. Blocks were defined according to the default method of Haploview. The numbers in the boxes are the pairwise LD coefficient *r*^2^ between SNPs. *r*^2^ values of 1 represent complete LD; values greater than 0.8 represent strong evidence of LD; values of 0.2–0.8 represent modest LD; and values less than 0.2 represent negligible evidence of LD.

Among the 61 SNPs genotyped, three were excluded due to poor genotyping success rate (<5%). The remaining 58 SNPs with genotyping success rate >95% conformed to Hardy–Weinberg equilibrium expectations (*P* > 0.01). The distributions of the allele and genotype frequencies of these SNPs in controls and HSCR patients are shown in Table 1. The LD pattern of SNPs and haplotype block was plotted in Figure 1.

**TABLE 1:** Allele distribution and association analysis for 58 SNPs in HSCR patients and healthy controls.

CHR	BP	SNP	Gene	Reference allele	Alternative allele	Frequency^a^		*P*_Additive_	OR (95% CI)
						Case	Control		
1	116234694	rs41312690	*CASQ2*	A	C	0.05	0.04	0.234	1.24 (0.87–1.75)
1	116236581	rs3811008	*CASQ2*	G	A	0.91	0.89	0.114	1.23 (0.95–1.60)
1	116240026	rs6428677	*CASQ2*	A	G	0.33	0.32	0.875	1.01 (0.86–1.19)
1	116241211	rs6673301	*CASQ2*	A	G	0.85	0.85	0.908	1.01 (0.82–1.25)
1	116243380	rs7521023	*CASQ2*	G	A	0.33	0.31	0.205	1.11 (0.94–1.30)
1	116250612	rs6684209	*CASQ2*	A	G	0.26	0.24	0.433	1.07 (0.90–1.28)
1	116260604	rs2997742	*CASQ2*	G	A	0.40	0.38	0.242	1.10 (0.94–1.28)
1	116283343	rs9428083	*CASQ2*	G	A	0.78	0.77	0.411	1.08 (0.90–1.30)
1	116284997	rs7554304	*CASQ2*	A	G	0.90	0.89	0.250	1.16 (0.90–1.49)
1	116302923	rs9428225	*CASQ2*	A	G	0.45	0.43	0.446	1.06 (0.91–1.24)
1	116310937	rs10801999	*CASQ2*	G	A	0.93	0.93	0.855	1.03 (0.77–1.38)
1	116310967	rs4074536	*CASQ2*	A	G	0.50	0.48	0.307	1.08 (0.93–1.26)
1	116316520	rs17504277	*CASQ2*	C	A	0.34	0.33	0.589	1.05 (0.89–1.23)
1	116320833	rs10923445	*CASQ2*	G	A	0.40	0.40	0.964	1.00 (0.85–1.16)
3	27526516	rs6771541	*SLC4A7,EOMES*	G	A	0.90	0.90	0.918	1.01 (0.79–1.30)
3	27529889	rs9871261	*SLC4A7,EOMES*	C	A	0.87	0.85	0.264	1.13 (0.91–1.41)
3	27535796	rs9836685	*SLC4A7,EOMES*	A	G	0.87	0.86	0.700	1.05 (0.84–1.31)
3	27537909	rs13082711	*SLC4A7,EOMES*	G	A	0.05	0.05	0.357	1.17 (0.84–1.64)
3	27542500	rs7635193	*SLC4A7,EOMES*	G	A	0.87	0.86	0.711	1.04 (0.84–1.30)
3	27546886	rs113102807	*SLC4A7,EOMES*	G	A	0.94	0.94	0.667	1.07 (0.78–1.48)
3	27548900	rs820430	*SLC4A7,EOMES*	G	A	0.68	0.68	0.742	1.03 (0.87–1.21)
3	27562613	rs2643825	*SLC4A7,EOMES*	G	A	0.05	0.05	0.482	1.13 (0.80–1.59)
3	27588131	rs34085137	*SLC4A7,EOMES*	A	G	0.05	0.04	0.405	1.17 (0.81–1.69)
3	27595912	rs17019924	*SLC4A7,EOMES*	A	G	0.79	0.79	0.777	1.03 (0.85–1.24)
3	27601508	rs9851320	*SLC4A7,EOMES*	A	C	0.21	0.18	0.042	1.22(1.01–1.47)
3	27602032	rs9852113	*SLC4A7,EOMES*	A	G	0.14	0.13	0.607	1.06 (0.85–1.32)
3	27608341	rs73055818	*SLC4A7,EOMES*	A	C	0.04	0.04	0.529	1.13 (0.77–1.66)
3	27612399	rs12639253	*SLC4A7,EOMES*	G	A	0.62	0.61	0.853	1.02 (0.87–1.19)
3	27614316	rs9854207	*SLC4A7,EOMES*	C	A	0.42	0.42	0.894	1.01 (0.87–1.18)
3	27615796	rs2642925	*SLC4A7,EOMES*	G	A	0.12	0.09	0.007	1.41 (1.10–1.79)
3	27639940	rs2643844	*SLC4A7,EOMES*	C	A	0.06	0.04	0.065	1.37 (0.99–1.91)
3	27646114	rs2643823	*SLC4A7,EOMES*	A	G	0.38	0.37	0.890	1.01 (0.86–1.18)
3	27652791	rs55966019	*SLC4A7,EOMES*	A	G	0.03	0.03	0.540	1.14 (0.74–1.77)
3	27659933	rs76747642	*SLC4A7,EOMES*	A	G	0.94	0.94	0.901	1.02 (0.74–1.42)
3	27680063	rs8179938	*SLC4A7,EOMES*	A	G	0.61	0.59	0.270	1.09 (0.94–1.28)
3	27702442	rs3856680	*SLC4A7,EOMES*	A	G	0.64	0.64	0.939	1.01 (0.86–1.18)
3	27721198	rs6764447	*SLC4A7,EOMES*	G	A	0.72	0.68	0.088	1.16 (0.98–1.37)
3	27726951	rs2642935	*SLC4A7,EOMES*	A	G	0.97	0.96	0.358	1.22 (0.80–1.87)
3	171314225	rs10936694	*PLD1*	A	G	0.77	0.76	0.829	1.02 (0.85–1.22)
3	171362785	rs2287579	*PLD1*	A	G	0.05	0.03	0.004	1.71 (1.18–2.46)
3	171404478	rs2290480	*PLD1*	A	C	0.14	0.12	0.048	1.25 (1.01–1.56)
3	171409603	rs7649974	*PLD1*	G	A	0.34	0.31	0.041	1.18 (1.01–1.39)
3	171449100	rs6773529	*PLD1*	G	A	0.58	0.57	0.752	1.03 (0.88–1.20)
3	171489402	rs9860368	*PLD1*	C	A	0.15	0.12	0.076	1.22 (0.98–1.52)
3	171495180	rs13098743	*PLD1*	A	G	0.04	0.04	0.872	1.03 (0.69–1.54)
3	171506951	rs6767600	*PLD1*	C	A	0.65	0.65	0.610	1.04 (0.89–1.22)
3	171511355	rs12632766	*PLD1*	G	A	0.75	0.71	0.038	1.20 (1.01–1.42)
3	171519793	rs13321742	*PLD1*	G	A	0.70	0.69	0.592	1.05 (0.89–1.23)
3	171529101	rs4894773	*PLD1*	A	G	0.65	0.64	0.823	1.02 (0.87–1.19)
10	43191350	rs138127971	*LINC01518,LOC283028*	G	A	0.05	0.03	0.021	1.52 (1.06–2.18)
10	43193161	rs2484304	*LINC01518,LOC283028*	G	A	0.98	0.96	0.028	1.71 (1.05–2.79)
10	43197926	rs7898080	*LINC01518,LOC283028*	A	G	0.95	0.92	0.002	1.65 (1.19–2.29)
10	43211650	rs12265862	*LINC01518,LOC283028*	A	G	0.27	0.19	7.68 ×10^−7^	1.57 (1.32–1.87)
10	43224684	rs11239753	*LINC01518,LOC283028*	A	G	0.05	0.03	0.006	1.71 (1.17–2.51)
10	43224701	rs10508868	*LINC01518,LOC283028*	G	A	0.05	0.03	0.011	1.59 (1.11–2.27)
10	43225728	rs17153309	*LINC01518,LOC283028*	A	C	0.28	0.19	1.13 ×10^−7^	1.60 (1.34–1.90)
10	43240428	rs1414027	*LINC01518,LOC283028*	A	T	0.75	0.68	3.92 ×10^−5^	1.43 (1.20–1.70)
10	43253587	rs624804	*LINC01518,LOC283028*	A	G	0.43	0.37	0.001	1.29 (1.11–1.50)

*^*a*^Reference allele frequency.*

None of the 14 SNPs in the *CASQ2* gene region showed significant association with HSCR susceptibility. Two tag SNPs located between *SLC4A7* and *EOMES* on 3p24.1 showed a suggestive significant association (Table 1): rs2642925 [odds ratio (OR) = 1.41, 95% confidence interval (95% CI) = 1.10–1.79; *P*_Additive_ = 0.007; Table 1] and the previously associated SNP rs9851320 (OR = 1.22, 95% CI = 1.01–1.47; *P*_Additive_ = 0.042; Table 1). A coding SNP, rs2287579, in *PLD1* showed a suggestive association with HSCR susceptibility (OR = 1.71, 95% CI = 1.18–2.46; *P*_Additive_ = 0.004; Table 1). This SNP resulted in an amino acid substitution from a valine to methionine (p.Val > Met) at position 820 of PLD1 protein isoform a. The previously associated *PLD1* SNP rs12632766 showed a suggestive significance (OR = 1.20, 95% CI = 1.01–1.42, *P*_Additive_ = 0.038; Table 1). Two new SNPs, rs2290480 and rs7649974, also showed suggestive association with HSCR susceptibility, *P*_Additive_ = 0.048 and 0.041, respectively. However, none of the *P* values of SNPs in 3p24.1 and the *PLD1* region could reach the study-wide significance (*P* > 0.00086).

All the nine tag SNPs located between *LINC01518* and *LOC283028* on 10q11.21 demonstrated a suggestive association (*P* < 0.05), and three of them met the Bonferroni multiple testing corrected *P* value threshold (*P* < 0.00086). The top SNP was rs17153309 (OR = 1.60, 95% CI = 1.34–1.90; *P*_Additive_ = 1.13 × 10^–7^; Table 1). Rs1414027 was reported to exhibit an association with HSCR risk in the previous study (Tang et al., 2018), and it was the third most associated SNP in the current data, with a *P*_Additive_ value of 3.92 × 10^–5^ (OR = 1.43, 95% CI = 1.20–1.70). Nine SNPs genotyped in this region were in low to moderate LD (Figure 1D). No significant SNP-SNP interaction was detected among the SNPs genotyped.

Furthermore, we applied stepwise conditional regression analysis to determine which SNPs in the *LINC01518*-*LOC283028* region confer independent risks on HSCR. When conditioning on the top SNP, rs17153309, rs1414027 showed the most significant independent evidence of association (conditional *P* = 4.73 × 10^–5^; OR = 1.44, 95% CI = 1.21–1.72). Similarly, rs17153309 was significantly associated with HSCR when conditioning on rs1414027 (*P* = 1.70 × 10^–7^; OR = 1.60, 95% CI = 1.34–1.90). Therefore, it was obvious these two SNPs represent independent associations. When conditioning on rs17153309 and rs1414027, no SNPs showed significant independent associations (conditional *P* > 0.00086). These results suggest that independent association signals existed in the *LINC01518*-*LOC283028* region and the combination of the above two SNPs could explain the majority of risk for this region.

In addition, considering that the clinical manifestations, surgical options, and post-operation complications differ significantly between S-HSCR, L-HSCR, and TCA patients, we performed association analysis stratified by clinical subtype and did not find SNPs associated with a specific subtype (Supplementary Table S3).

### Functional Annotation

Putative function of associated SNPs was annotated by HaploReg (Table 2). At the 10q11.21 locus, the six associated SNPs altered the sequences of regulatory motifs (Table 2). Additionally, rs624804 maps to a DNase I hypersensitivity site (Table 2). Two associated SNPs (rs1414027 and rs624804) at 10q11.21 were eQTLs of nearby genes (Table 3). Rs1414027 was an eQTL of *ZNF33B* in the small intestine (*P* = 4.60 × 10^–3^; PP.H4 = 0.853), transverse colon (*P* = 3.40 × 10^–9^; PP.H4 = 0.999), and sigmoid colon (*P* = 9.30 × 10^–5^; PP.H4 = 0.682). Rs624804 was an eQTL of *LINC00839* in the esophagus (*P*_muscularis_ = 6.50 × 10^–12^, PP.H4 = 0.777; *P*_mucosa_ = 2.80 × 10^–9^, PP.H4 = 0.992; *P*_Gastroesophageal Junction_ = 9.30 × 10^–10^, PP.H4 = 0.995), while rs624804 was also an eQTL of *RP11-313J2.1* in the stomach (*P* = 1.20 × 10^–11^; PP.H4 = 0.999).

**TABLE 2:** Functional annotation of positive SNPs associated with HSCR using data from Haploreg and ENCODE.

SNP	Region	Promoter histone marks^a^	Enhancer histone marks^b^	DNAse^c^	Motifs changed^d^
rs7898080	*LINC01518*-*LOC283028*				CACD, RREB-1, VDR
rs12265862	*LINC01518*-*LOC283028*				Gfi1, Gfi1b, Mef2
rs11239753	*LINC01518*-*LOC283028*				Foxj1
rs17153309	*LINC01518*-*LOC283028*				BCL
rs1414027	*LINC01518*-*LOC283028*				Barx1, Hdx, NF-Y
rs624804	*LINC01518*-*LOC283028*			MUS	GATA, Maf, Myf

*^*a*^Evidence of local H3K4Me1 and H3K27Ac modification. ^*b*^Evidence of local H3K4Me3 modification (cell lines/types: if >3, only the number is included). ^*c*^Evidence of chromatin hypersensitivity to DNase. ^*d*^Evidence of alteration in regulatory motifs. ENCODE, the Encyclopedia of DNA Elements.*

**TABLE 3:** The expression quantitative trait loci (eQTL) evidence of the positive SNPs.

SNP	Correlated gene	Tissues	eQTL *P* (GTEx)	PP.H4 (coloc)
rs1414027	*ZNF33B*	Small intestine–terminal ileum	4.6 × 10^–3^	0.853
rs1414027	*ZNF33B*	Colon–transverse	3.4 × 10^–9^	0.999
rs1414027	*ZNF33B*	Colon – sigmoid	9.3 × 10^–5^	0.682
rs624804	*LINC00839*	Esophagus – muscularis	6.5 × 10^–12^	0.777
rs624804	*LINC00839*	Esophagus – mucosa	2.8 × 10^–9^	0.992
rs624804	*LINC00839*	Esophagus – gastroesophageal junction	9.3 × 10^–10^	0.995
rs624804	*RP11-313J2.1*	Stomach	1.2 × 10^–11^	0.999

## Discussion

Four common SNPs at different chromosome regions were found to be associated with HSCR susceptibility in an earlier study (Tang et al., 2018). In the current study, we genotyped 61 SNPs to validate the association and fine-map the susceptibility loci in the Han Chinese population. Our results indicated a suggestive association of HSCR susceptibility with variants in *SLC4A7*-*EOMES* and *PLD1*, and significant association with variants in the *LINC01518*-*LOC283028* regions.

None of the 14 tag SNPs in the *CASQ2* region showed any association with HSCR susceptibility. The previously associated *CASQ2* SNP, rs9428225, also displayed no significance in our sample-set. The risk allele A of rs9428225 was 0.472 in cases and 0.356 in controls in the previous study on Chinese and Vietnamese populations, as shown in Supplementary Table S4 (Tang et al., 2018). In the current sample-set of the Han Chinese population, the frequencies of rs9428225 A allele were 0.448 in cases and 0.434 in controls. Thus, the rs9428225 A allele had the same effect direction in the current sample as in the previous data, though the difference of allele frequency between cases and controls was not significant. Owing to the modest effect of this locus, further studies are needed to validate this association.

In the intergenic region between *SLC4A7* and *EOMES* on 3p24.1, the previously associated SNP rs9851320 showed a suggestive association in our samples. The frequencies of rs9851320 minor allele A were 0.213 in cases and 0.181 in controls in the current Han Chinese population, which followed the same trend as that of as previous data on Chinese and Vietnamese populations, with 0.248 in cases and 0.164 in controls (Tang et al., 2018). The frequency of rs9851320 minor allele A is 0.186 in the South Han Chinese data in the 1000 genomes project, which is close to that observed in the current sample set (0.181) (Supplementary Table S4). *SLC4A7* encodes a sodium bicarbonate cotransporter. The highest expression level of *SLC4A7* was detected in the duodenum [reads per kilobase per million mapped reads (RPKM), 27.5] and the small intestine (RPKM 8.8) from quantitative transcriptomics analysis (Fagerberg et al., 2014). *EOMES* is also known as T-brain gene 2 (*TBR2*) and in mammals, the transcription factor Eomes/Tbr2 regulates neurogenesis in the cortical subventricular zone (Arnold et al., 2008). In addition, Eomes was indispensable for hippocampal lineage progression from neural stem cells to intermediate progenitors and neurons (Hodge et al., 2012). Without Tbr2, intermediate neuronal progenitors were depleted in spite of augmented neural stem cell proliferation, and neurogenesis ceased due to the failure of neuronal differentiation (Hodge et al., 2012). Hirschsprung’s disease is caused by the developmental defect of the enteric neuron system (ENS; Heuckeroth, 2018). Therefore, *SLC4A7* and *EOMES* may play a role in the development of ENS and this could provide a link to the pathogenesis of HSCR. However, further studies are required to validate the association at these loci with HSCR.

We identified a non-synonymous SNP, rs2287579, (p.Val 820 Met) in *PLD1* associated with HSCR susceptibility with suggestive evidence. The minor allele A frequency of previously associated rs12632766 was 0.251 in cases and 0.286 in controls in the current Han Chinese population. The frequencies were 0.196 and 0.293 in cases and controls, respectively, in the previous study of Chinese and Vietnamese populations, as shown in Supplementary Table S4 (Tang et al., 2018). The effect direction of current data was consistent with previous data that rs12632766 minor allele A exhibited a protective effect in HSCR susceptibility, although the association could not reach the significance threshold. *PLD1* encodes a phosphatidylcholine-specific phospholipase, which could catalyze the hydrolysis of phosphatidylcholine to produce phosphatidic acid and choline. This enzyme may play a crucial role in signal transduction. PLD1 signaling pathway genes might account for disease severity of HSCR (Tang et al., 2018). Notably, the BACE1-APP-BACE2 pathway may be an important pathway underlying the etiology of HSCR (Tang et al., 2018). *PLD1* was found to be involved in Abeta production via regulating amyloid precursor protein (APP) metabolism and trafficking (Cai et al., 2006a, b). All this evidence suggests that *PLD1* may play a role in the pathogenesis of HSCR. However, the association of *PLD1* with *HSCR* needs to be confirmed in additional samples.

Variants located between *LINC01518* and *LOC283028* on 10q11.21 showed significant associations in the current study. The previously associated SNP, rs1414027, was successfully replicated in our samples. Two SNPs represented independent association signals and accounted for the association signal of the locus. Both *LINC01518* and *LOC283028* are long non-coding RNAs (lncRNAs). Recent studies have revealed that lncRNAs played an important role in the pathogenesis of HSCR. Dysregulated lncRNAs were found in HSCR tissues, which suggested their probable use as novel diagnostic biomarkers for HSCR (Shen et al., 2016). Notably, the associated SNP, rs1414027, was an eQTL of *ZNF33B* in multiple tissues, and rs624804 was an eQTL of *LINC00839* and *RP11-313J2.1*. However, no colocalized signals were observed convincingly across all digestive tract tissues, emphasizing the tissue specific effects of disease causal variants. *ZNF33B* encodes a member of the zinc finger family of proteins and was expressed in the colon and small intestine tissues, implicating its potential role in the development of gut tissues (Fagerberg et al., 2014). *LINC00839* was highly expressed in the colon, esophagus, small intestine, and stomach (Fagerberg et al., 2014). Since all the above-mentioned genes may be potential susceptibility genes, further functional studies are needed to clarify the causal gene in this region.

The relatively small sample size limits the current study. Under the assumption of 0.0002 disease prevalence, the study-wide significance level, and odds ratios of 1.3/1.6 for heterozygotes/rare homozygotes, 420 cases and 1,665 controls could achieve 38.8, 24.7, 39.1, and 37.5% statistical power for rs9428225 (MAF = 0.43), rs9851320 (MAF = 0.18), rs12632766 (MAF = 0.39), and rs1414027 (MAF = 0.32). However, the statistical power would be much lower with the threshold of *P*-value, calculated using a strict Bonferroni correction for multiple comparisons. Much larger sample sizes are required to achieve an adequate statistical power.

In conclusion, we replicated the association of variants of *LINC01518*-*LOC283028* at 10q11.21 with HSCR susceptibility. Our fine-mapping also revealed new associated variants, and two independent SNPs accounting for the association of the *LINC01518*-*LOC283028* region with HSCR. Additionally, eQTL analysis indicated that these disease-associated SNPs influenced the expression levels of nearby genes. These findings provide new insights toward understanding the pathogenesis of HSCR.

## Data Availability Statement

The original contributions presented in the study are publicly available. This data can be found at https://www.ncbi.nlm.nih.gov/bioproject/PRJNA597838.

## Ethics Statement

The studies involving human participants were reviewed and approved by The Ethics Committee of the Xin Hua Hospital affiliated to Shanghai Jiao Tong University School of Medicine. Written informed consent to participate in this study was provided by the participants’ legal guardian/next of kin.

## Author Contributions

WC and XC conceived the study. M-RB, Y-JL, X-XY, Z-LW, W-JW, H-LS, Y-MG, W-WY, and B-LG conducted the experiment. W-BN and XC participated in data analysis and figure preparation and drafted the manuscript. All authors read and approved the final manuscript.

## Conflict of Interest

The authors declare that the research was conducted in the absence of any commercial or financial relationships that could be construed as a potential conflict of interest.

## Footnotes

1 http://gvs.gs.washington.edu/GVS150/

2 http://zzz.bwh.harvard.edu/gpc/
