# Supplementary material for: Association of Variants in PLD1, 3p24.1, and 10q11.21 Regions With Hirschsprung’s Disease in Han Chinese Population
Source: Front Genet. 2020 Jul 10;11:738. doi: 10.3389/fgene.2020.00738 (PMC7381268; doi:10.3389/fgene.2020.00738)
Supplement: Supplementary file 1 [file Data_Sheet_1.docx]

SUPPLEMENTARY MATERIAL

Wei-Bo Niu^†^, Mei-Rong Bai^†^, Huan-Lei Song, Yan-Jiao Lu, Wen-Jie Wu, Yi-Ming Gong, Xian-Xian Yu, Zhi-Liang Wei, Wen-Wen Yu, Bei-Lin Gu, Wei Cai* and Xun Chu*

Supplementary Table S1. SNPs genotyping primers

| SNP | Gene | Forward Primer | Reverse Primer | Probe 1 | Probe 2 |
| --- | --- | --- | --- | --- | --- |
| rs41312690 | CASQ2 | TTGAGGCTTCACACAGAGCC | TTCTGATCCCACCCCTCTGC | TGCGACTAAGAACGCTATCAG GATGGCATGGAGGCCAGG | CAAGTGATCCGAGAGGTTGAAC GATGGCATGGAGGCCAGT |
| rs3811008 | CASQ2 | ACATCACCAGGGAAGAGAGCA | AGACAGGATTTCATTTGCCTTATATGT | TGCGACTAAGAACGCTATCAG AACACTCAATGCTCTAATTTTAATAAC | CAAGTGATCCGAGAGGTTGAAC AACACTCAATGCTCTAATTTTAATAAT |
| rs6428677 | CASQ2 | AGGGGTCCCAGAGGAGTTTG | TGGAACATTCGCTTACTTTTCATCA | TGCGACTAAGAACGCTATCAG AGTTTGCCATTTAATTAAAGCCTACT | CAAGTGATCCGAGAGGTTGAAC AGTTTGCCATTTAATTAAAGCCTACC |
| rs6673301 | CASQ2 | AGTAAACCAGCAAGTGGAAAGGT | TGAATTCCCAAGCCACTCAGG | TGCGACTAAGAACGCTATCAG GGGTTGATGTGGGAGGTGAA | CAAGTGATCCGAGAGGTTGAAC GGGTTGATGTGGGAGGTGAG |
| rs7521023 | CASQ2 | AGCCCAGGGAAGACACATGA | AAGTTTGCTTCCAAGGCTGAG | TGCGACTAAGAACGCTATCAG ATCAGTTGGGATCCCTCCTCT | CAAGTGATCCGAGAGGTTGAAC ATCAGTTGGGATCCCTCCTCC |
| rs6684209 | CASQ2 | AGGAACGTAGTAGGTAAGTTCTTGT | CAAGTGTTGTGGAGGGACAGG | TGCGACTAAGAACGCTATCAG CCATAAGAAAACAACAGTGCTCCG | CAAGTGATCCGAGAGGTTGAAC CCATAAGAAAACAACAGTGCTCCA |
| rs2997742 | CASQ2 | AGCTGGAGGGATGCAAAGGA | CGCTTCCACACCTCTCCCTA | TGCGACTAAGAACGCTATCAG GGACTCAGAAGCTGGAATGGA | CAAGTGATCCGAGAGGTTGAAC GGACTCAGAAGCTGGAATGGG |
| rs9428083 | CASQ2 | ATGGCGAGTTTGCAGCTGAT | GCCAGGTTCTCCAGCTTGTC | TGCGACTAAGAACGCTATCAG GTGGAGTTCCTCTTGGATGTAAGT | CAAGTGATCCGAGAGGTTGAAC GTGGAGTTCCTCTTGGATGTAAGC |
| rs7554304 | CASQ2 | CATTGGCCAGTGTCCTGCTG | TGGCCTCTATCAAGTTGTGCC | TGCGACTAAGAACGCTATCAG GGATTTTGTCTTGAGCAAAACCA | CAAGTGATCCGAGAGGTTGAAC GGATTTTGTCTTGAGCAAAACCG |
| rs9428225 | CASQ2 | GGACCCCAGACCAGTAGCAA | TGTCACAGTGCTTTGCAAACTT | TGCGACTAAGAACGCTATCAG GCATTTAAATGAGATCCCTGTGTCG | CAAGTGATCCGAGAGGTTGAAC GCATTTAAATGAGATCCCTGTGTCA |
| rs10801999 | CASQ2 | CAGACAGCATGCCCTTTGGT | CCATGAGCCGGTGTCTTCAG | TGCGACTAAGAACGCTATCAG CCCTTTGGTTACTTACCTCAAGCAC | CAAGTGATCCGAGAGGTTGAAC CCCTTTGGTTACTTACCTCAAGCAT |
| rs4074536 | CASQ2 | CCATGAGCCGGTGTCTTCAG | CAGACAGCATGCCCTTTGGT | TGCGACTAAGAACGCTATCAG CCGGTGTCTTCAGATAAGGTCG | CAAGTGATCCGAGAGGTTGAAC CCGGTGTCTTCAGATAAGGTCA |
| rs17504277 | CASQ2 | TCCTAAGGAGAGATATTGAGAGATGGT | CCATGAGGGAGCTGGGACAT | TGCGACTAAGAACGCTATCAG AAAGAAAGGTCAAAAACGTAGGAGAC | CAAGTGATCCGAGAGGTTGAAC AAAGAAAGGTCAAAAACGTAGGAGAA |
| rs10923445 | CASQ2 | GCCAGCAGATTTGGTGTCCA | AAGATTCCTCAGCCCTGCCA | TGCGACTAAGAACGCTATCAG ACTGTCTTCTCTTTGTGTCCTCAC | CAAGTGATCCGAGAGGTTGAAC ACTGTCTTCTCTTTGTGTCCTCAT |
| rs6771541 | SLC4A7,EOMES | TGTACACTTGACCAGGTGTCCT | GGCTGGTGCAAGACTCTGT | TGCGACTAAGAACGCTATCAG AGCACCACGACAGCTGCT | CAAGTGATCCGAGAGGTTGAAC GCACCACGACAGCTGCC |
| rs9871261 | SLC4A7,EOMES | GCACTCCAGCCTGGACAG | GAACAATTCTCTATAGGCCAAGGT | TGCGACTAAGAACGCTATCAG TGGTTTCACACTTTTCAAAATACG | CAAGTGATCCGAGAGGTTGAAC TGGTTTCACACTTTTCAAAATACA |
| rs9836685 | SLC4A7,EOMES | CAAACCATATCACACAGCATCC | GGAATTCAGCAACAGACATAC | TGCGACTAAGAACGCTATCAG TTCCATTTTTTTATGAATATACACCAG | CAAGTGATCCGAGAGGTTGAAC TTCCATTTTTTTATGAATATACACCAA |
| rs13082711 | SLC4A7,EOMES | ACAAGGACATCCAGACTGGC | CTGTCCCACACCTATGTTCTCT | TGCGACTAAGAACGCTATCAG CCAGACTGGCCCTCATCA | CAAGTGATCCGAGAGGTTGAAC CAGACTGGCCCTCATCG |
| rs7635193 | SLC4A7,EOMES | TGTAGCCTGTCAGCACCACTAC | AGTCATCTGAAGCCCAGGTC | TGCGACTAAGAACGCTATCAG TGGCCATAATATCTACAGAGGCT | CAAGTGATCCGAGAGGTTGAAC TGGCCATAATATCTACAGAGGCC |
| rs113102807 | SLC4A7,EOMES | GCCAACAAGGCAGGAGAA | GTCTCACTCTATCGCCCAGG | TGCGACTAAGAACGCTATCAG CTTGAACCCTGGGAGGTGA | CAAGTGATCCGAGAGGTTGAAC TTGAACCCTGGGAGGTGG |
| rs820430 | SLC4A7,EOMES | AGATTCTCCAAAGTTTTGATGCT | AACAAGCCCTTCAGGAAGTG | TGCGACTAAGAACGCTATCAG AAAGTTTTGATGCTTTAGCTATATTCC | CAAGTGATCCGAGAGGTTGAAC AAAGTTTTGATGCTTTAGCTATATTCT |
| rs2643825 | SLC4A7,EOMES | CCTTCCAGGATGATCCACA | GAGTTCCAGTGGGAGAGACAGT | TGCGACTAAGAACGCTATCAG CATTTTCTTTCTGTCCTTTGTCC | CAAGTGATCCGAGAGGTTGAAC TCATTTTCTTTCTGTCCTTTGTCT |
| rs34085137 | SLC4A7,EOMES | ATGATCTGTGCCCCTCCA | TGCCAATGTTTGTCACTAGTCTT | TGCGACTAAGAACGCTATCAG AAAAGGAAGCCAGAAAGCG | CAAGTGATCCGAGAGGTTGAAC AAAAAGGAAGCCAGAAAGCA |
| rs17019924 | SLC4A7,EOMES | CAACCAGCAAATGCAAGGA | CTGACAGAGGAGGTCTTTGGA | TGCGACTAAGAACGCTATCAG CAAGGAGAATCTGCTTAACTGTG | CAAGTGATCCGAGAGGTTGAAC GCAAGGAGAATCTGCTTAACTGTA |
| rs9851320 | SLC4A7,EOMES | TCAATCATACCGCAGCTGAG | TCCGGATTTGGATGAAATG | TGCGACTAAGAACGCTATCAG CTAGTGCTCTGCAGCTGGATA | CAAGTGATCCGAGAGGTTGAAC TAGTGCTCTGCAGCTGGAC |
| rs9852113 | SLC4A7,EOMES | CAGGGTTTCGTCTACCTTCCT | TTACTCTGTTGTGCGGGTG | TGCGACTAAGAACGCTATCAG CGTCTACCTTCCTCTCTTCCAT | CAAGTGATCCGAGAGGTTGAAC GTCTACCTTCCTCTCTTCCAC |
| rs73055818 | SLC4A7,EOMES | GGCTTTGTTTCTTAGAGCAGG | CCCGAGGACTCCTATCTATTTG | TGCGACTAAGAACGCTATCAG TTTCTTAGAGCGGGTTCAGG | CAAGTGATCCGAGAGGTTGAAC TGTTTCTTAGAGCGGGTTCAGT |
| rs12639253 | SLC4A7,EOMES | CCACAGTTTCAGAAACTCCCT | ACTTTCCATGTCAGTGTGCTCT | TGCGACTAAGAACGCTATCAG CAGGCTCCATGATGCAGAT | CAAGTGATCCGAGAGGTTGAAC CAGGCTCCATGATGCAGAC |
| rs9854207 | SLC4A7,EOMES | AACTTGCTTAGGAGGTAGAGAGG | TCTCTGGACTTCTCTGTTACAGGA | TGCGACTAAGAACGCTATCAG AGAACATGGTGGGGTGGAT | CAAGTGATCCGAGAGGTTGAAC AGAACATGGTGGGGTGGAG |
| rs2642925 | SLC4A7,EOMES | TGTGCTGAGAAGGAGCAAAC | GGGTACGGAGCATCTACACAA | TGCGACTAAGAACGCTATCAG AGAAGGAGCAAACATTGGTG | CAAGTGATCCGAGAGGTTGAAC TGAGAAGGAGCAAACATTGGTA |
| rs2643844 | SLC4A7,EOMES | TCCATGGACAACCACTTTTG | CTCAAAAAAAAAGGCAGGCAC | TGCGACTAAGAACGCTATCAG AAACGGTATCAACCAGGGG | CAAGTGATCCGAGAGGTTGAAC ATATAAACGGTATCAACCAGGGT |
| rs2643823 | SLC4A7,EOMES | TTAACTGTTCATCACCTAAACAAGTT | ACATGAAGCCCCTAATGACTCT | TGCGACTAAGAACGCTATCAG TCACCTAAACAAGTTTATTGGGC | CAAGTGATCCGAGAGGTTGAAC ATCACCTAAACAAGTTTATTGGGT |
| rs55966019 | SLC4A7,EOMES | CTTGGTCATGTGATGGCGTT | CTGCAGAAAGCTGCAGAATC | TGCGACTAAGAACGCTATCAG CCAAATATAACATCCATCTGTCCG | CAAGTGATCCGAGAGGTTGAAC CAAATATAACATCCATCTGTCCA |
| rs76747642 | SLC4A7,EOMES | TCGAGGGCTCACTATGTAGAAG | TGTGTGTTCTTCGAAGGGAG | TGCGACTAAGAACGCTATCAG CCTAGTGTTAACTACCATGGGCG | CAAGTGATCCGAGAGGTTGAAC CTAGTGTTAACTACCATGGGCA |
| rs8179938 | SLC4A7,EOMES | GCTGCTCACTGTCATACGAAG | TCACAGTCACTTCACTTTAATGAGA | TGCGACTAAGAACGCTATCAG AAGAAGGATCCATGCCACAT | CAAGTGATCCGAGAGGTTGAAC AAGAAGGATCCATGCCACAC |
| rs3856680 | SLC4A7,EOMES | ATCTCGTTGAGGAAAGCTGG | TAGTGTTTACAGCAGGTTGG | TGCGACTAAGAACGCTATCAG TCCCCACTGCCACACCTA | CAAGTGATCCGAGAGGTTGAAC TCCCCACTGCCACACCTG |
| rs6764447 | SLC4A7,EOMES | GGTTCAGTATTAGCTGCAGTCTCA | AACAGTAGTTCCCCCTTATCGTC | TGCGACTAAGAACGCTATCAG ATTAGCTGCAGTCTCATCCACTA | CAAGTGATCCGAGAGGTTGAAC TTAGCTGCAGTCTCATCCACTG |
| rs2642935 | SLC4A7,EOMES | GACTTTCTTGGAGAATACAGGGA | AAGACAACCCAAGGATAGAGAGA | TGCGACTAAGAACGCTATCAG ACAGGGATAAAATTCTTCCCTCT | CAAGTGATCCGAGAGGTTGAAC AGGGATAAAATTCTTCCCTCC |
| rs360401 | PLD1 | TTGGAGAGGGTGATTCCAAC | CACTGCAATGTGTTCTCTAC | TGCGACTAAGAACGCTATCAG CCTCCACCATAAGGTCCC | CAAGTGATCCGAGAGGTTGAAC GCCTCCACCATAAGGTCCT |
| rs10936694 | PLD1 | GAGCACTGGAGTATGTTTGGC | CACAGCCTCTTCTAGGCACTTC | TGCGACTAAGAACGCTATCAG CCAGTTATAAAGGTAGTCAATGAACTA | CAAGTGATCCGAGAGGTTGAAC CAGTTATAAAGGTAGTCAATGAACTG |
| rs2287579 | PLD1 | AATGTCTCCTTCGAACCCTG | CTGACCTTTGCAGGGAAAAC | TGCGACTAAGAACGCTATCAG CCCTGGCAGAAGTGGTATCAT | CAAGTGATCCGAGAGGTTGAAC CCTGGCAGAAGTGGTATCAC |
| rs4894707 | PLD1 | ATGACAACAGATCGTGGCAG | TTCTCTATCTGCTGAGGTTACCA | TGCGACTAAGAACGCTATCAG GCAGGAGACCTTTTGATTTAGA | CAAGTGATCCGAGAGGTTGAAC GCAGGAGACCTTTTGATTTAGG |
| rs2290480 | PLD1 | TAAGGGAGTTCTGCCACTTCA | AACTCTTTCACCCGTCCAGT | TGCGACTAAGAACGCTATCAG CCACTTCACAAATTACTTACCAGC | CAAGTGATCCGAGAGGTTGAAC GCCACTTCACAAATTACTTACCAGA |
| rs7649974 | PLD1 | TTGCCAATATCTAAAACCTATCAA | TTTAAGCCTGCTGGAGGTT | TGCGACTAAGAACGCTATCAG AACCTATCAATAAATATAACCCAGTCAC | CAAGTGATCCGAGAGGTTGAAC AACCTATCAATAAATATAACCCAGTCAT |
| rs6773529 | PLD1 | AGACCTCTGCCTCATATTTGTG | GCTTTTTACAGACAGTAACGCA(1) GCTTTTTTACAGACAGTAACGCA (2) | TGCGACTAAGAACGCTATCAG TGTGAAATGAGATTGGAATTTACG | CAAGTGATCCGAGAGGTTGAAC TGTGAAATGAGATTGGAATTTACA |
| rs9860368 | PLD1 | CAAAGAGAACTCTCACTCCCTTC | GCAGACTGTATCCCCTTGCT | TGCGACTAAGAACGCTATCAG CCCTCAGACACTGCAATAAAGT | CAAGTGATCCGAGAGGTTGAAC CCCTCAGACACTGCAATAAAGG |
| rs13098743 | PLD1 | AAGGCAGAGGTTTCGCTTAG | CATCAAGGGCTTGTTCCTG | TGCGACTAAGAACGCTATCAG CGCTTAGAGCTGCTTGCA | CAAGTGATCCGAGAGGTTGAAC GCTTAGAGCTGCTTGCG |
| rs6767600 | PLD1 | AGAAAATGGTGGTCAGGGC | GCTCTTTGAATAGGATGCCC | TGCGACTAAGAACGCTATCAG ACTCACCAATGCACAGGATG | CAAGTGATCCGAGAGGTTGAAC AACTCACCAATGCACAGGATT |
| rs12632766 | PLD1 | CATAGGGCTTATGTTCAAGTAACTG | TCAGTTACCTGCAGTCAACCA | TGCGACTAAGAACGCTATCAG AATGGCTACAAAGTGCAAAAGTAGT | CAAGTGATCCGAGAGGTTGAAC TGGCTACAAAGTGCAAAAGTAGC |
| rs13321742 | PLD1 | TTCTGACCTCACAGATGTTGC | CGACAGGAGGCATTCACATAG | TGCGACTAAGAACGCTATCAG ATTTGGAACAAAGTGCTGCTC | CAAGTGATCCGAGAGGTTGAAC ATTTGGAACAAAGTGCTGCTT |
| rs4894773 | PLD1 | GCCAGTTCCAACCTCATTACA | ACGTTATTAGCAGTTTGGTGCA | TGCGACTAAGAACGCTATCAG CATCCCGCCAAAGCCAA | CAAGTGATCCGAGAGGTTGAAC ATCCCGCCAAAGCCAG |
| rs10900259 | LINC01518,LOC283028 | CATTCCAGCCTGGGTGACAG | AGGGTGGCCCTGGTAACATT | TGCGACTAAGAACGCTATCAG CACCGCCCCCACCCCTC | CAAGTGATCCGAGAGGTTGAAC CACCGCCCCCACCCCTA |
| rs138127971 | LINC01518,LOC283028 | TCCTAGCCCAGACCCACTTC | ATAGTGGCTTGTGGCACTGG | TGCGACTAAGAACGCTATCAG CCCAGACCCACTTCCTTCTAT | CAAGTGATCCGAGAGGTTGAAC CCCAGACCCACTTCCTTCTAC |
| rs2484304 | LINC01518,LOC283028 | CTGCTTCGTGGCTAATGCCT | CACCGGGTGGCATAGCTTTT | TGCGACTAAGAACGCTATCAG CCAAACCCACTTGCCTATCTGAT | CAAGTGATCCGAGAGGTTGAAC CCAAACCCACTTGCCTATCTGAC |
| rs7898080 | LINC01518,LOC283028 | CTGACCTTGTGATCTGCCCG | TCACGAAAATCAGGTCGGGT | TGCGACTAAGAACGCTATCAG GATTACAGGCGTGAACCACCG | CAAGTGATCCGAGAGGTTGAAC GATTACAGGCGTGAACCACCA |
| rs12265862 | LINC01518,LOC283028 | TTCACCCAACTCTGGAGTTTCT | CCCTGATCCGTAGAGAAGTGC | TGCGACTAAGAACGCTATCAG AACTCTGGAGTTTCTAAATCTGAGT | CAAGTGATCCGAGAGGTTGAAC AACTCTGGAGTTTCTAAATCTGAGC |
| rs11239753 | LINC01518,LOC283028 | ACCACACAGTGAGGCAAGTT | ATGCATACACGTGGCTTCCC | TGCGACTAAGAACGCTATCAG AGGAGCCGGGCTGAAAGC | CAAGTGATCCGAGAGGTTGAAC AGGAGCCGGGCTGAAAGT |
| rs10508868 | LINC01518,LOC283028 | GAGGGTGCCTTTTGGATTATGC | TCTAAGGAGCCGGGCTGAAA | TGCGACTAAGAACGCTATCAG GCTTCCCTGCTCCGTTTTGT | CAAGTGATCCGAGAGGTTGAAC GCTTCCCTGCTCCGTTTTGC |
| rs17153309 | LINC01518,LOC283028 | TCTCAGGTGACTTCAGGTGTTG | GCAGACATTTGCCCACTTCC | TGCGACTAAGAACGCTATCAG CAGGTGTTGAAACATAAAGCTTTT | CAAGTGATCCGAGAGGTTGAAC CAGGTGTTGAAACATAAAGCTTTG |
| rs1414027 | LINC01518,LOC283028 | GAGAAATGGCTGCTTTCTGG | AACGTGCCCTTGCTTTCC | TGCGACTAAGAACGCTATCAG CTGCAGGCAGGCAATGT | TGCGACTAAGAACGCTATCAG GCTGCAGGCAGGCAATGA |
| rs624804 | LINC01518,LOC283028 | GACTCAGCCAAGTGGCACAA | TGCCAGAGGTTTGAAGTCAGG | TGCGACTAAGAACGCTATCAG CTCTCTCATCTGAAAACAGCTGAC | CAAGTGATCCGAGAGGTTGAAC CTCTCTCATCTGAAAACAGCTGAT |

Supplementary Table S2. PCR cycling protocols

| Cycles | Temperature | Time |
| --- | --- | --- |
| Hot start | 95°C | 10 min |
| 1 | 95°C | 15 sec |
|  | 64°C | 45 sec |
|  | 72°C | 15 sec |
| 1 | 95°C | 15 sec |
|  | 63°C | 45 sec |
|  | 72°C | 15 sec |
| 1 | 95°C | 15 sec |
|  | 62°C | 45 sec |
|  | 72°C | 15 sec |
| 1 | 95°C | 15 sec |
|  | 61°C | 45 sec |
|  | 72°C | 15 sec |
| 30 | 95°C | 15 sec |
|  | 60°C | 45 sec |
|  | 72°C | 15 sec |

Supplementary Table S3. Association analysis Stratified by segment length of aganglionosis.

|  |  |  | |  |  | S-HSCR | | |  | L-HSCR | | |  | TCA | | |
| --- | --- | --- | --- | --- | --- | --- | --- | --- | --- | --- | --- | --- | --- | --- | --- | --- |
| CHR | SNP | | BP | A1 | F_U | F_A | *P* | OR (95%CI) |  | F_A | *P* | OR (95%CI) |  | F_A | *P* | OR (95%CI) |
| 1 | rs41312690 | | 116234694 | A | 0.04 | 0.05 | 0.1943 | 1.29(0.88- 1.88) |  | 0.04 | 0.9933 | 1.00( 0.40- 2.50) |  | 0.04 | 0.8468 | 0.89( 0.28- 2.86) |
| 1 | rs3811008 | | 116236581 | G | 0.89 | 0.91 | 0.1922 | 1.21(0.91- 1.61) |  | 0.89 | 0.9598 | 0.99( 0.55- 1.77) |  | 0.94 | 0.1939 | 1.82( 0.73- 4.52) |
| 1 | rs6428677 | | 116240026 | A | 0.32 | 0.33 | 0.877 | 1.01(0.85- 1.21) |  | 0.40 | 0.104 | 1.37( 0.94- 2.00) |  | 0.36 | 0.5208 | 1.17( 0.73- 1.86) |
| 1 | rs6673301 | | 116241211 | A | 0.85 | 0.85 | 0.8931 | 1.02(0.80- 1.29) |  | 0.88 | 0.4141 | 1.27( 0.72- 2.23) |  | 0.83 | 0.647 | 0.87( 0.48- 1.59) |
| 1 | rs7521023 | | 116243380 | G | 0.31 | 0.33 | 0.2973 | 1.10(0.92- 1.32) |  | 0.30 | 0.8782 | 0.97( 0.65- 1.45) |  | 0.31 | 0.9892 | 1.00( 0.61- 1.62) |
| 1 | rs6684209 | | 116250612 | A | 0.24 | 0.26 | 0.515 | 1.07(0.88- 1.30) |  | 0.30 | 0.1569 | 1.34( 0.89- 2.00) |  | 0.28 | 0.4415 | 1.22( 0.74- 2.00) |
| 1 | rs2997742 | | 116260604 | G | 0.38 | 0.40 | 0.2957 | 1.10(0.92- 1.30) |  | 0.38 | 0.9367 | 1.02( 0.69- 1.49) |  | 0.36 | 0.7633 | 0.93( 0.58- 1.49) |
| 1 | rs9428083 | | 116283343 | G | 0.77 | 0.78 | 0.5532 | 1.06(0.87- 1.30) |  | 0.78 | 0.8586 | 1.04( 0.67- 1.62) |  | 0.77 | 0.9924 | 1.00( 0.59- 1.71) |
| 1 | rs7554304 | | 116284997 | A | 0.89 | 0.90 | 0.3793 | 1.13(0.86- 1.50) |  | 0.88 | 0.7924 | 0.93( 0.52- 1.64) |  | 0.91 | 0.5235 | 1.29( 0.59- 2.82) |
| 1 | rs9428225 | | 116302923 | A | 0.43 | 0.45 | 0.5915 | 1.05(0.88- 1.24) |  | 0.37 | 0.1747 | 0.77( 0.52- 1.13) |  | 0.46 | 0.6301 | 1.12( 0.71- 1.75) |
| 1 | rs10801999 | | 116310937 | G | 0.93 | 0.93 | 0.9792 | 1.00(0.73- 1.39) |  | 0.90 | 0.2231 | 0.69( 0.37- 1.26) |  | 0.92 | 0.9027 | 0.95( 0.41- 2.20) |
| 1 | rs4074536 | | 116310967 | A | 0.48 | 0.49 | 0.4852 | 1.06(0.90- 1.26) |  | 0.45 | 0.5613 | 0.90( 0.62- 1.30) |  | 0.51 | 0.5162 | 1.16( 0.74- 1.82) |
| 1 | rs17504277 | | 116316520 | C | 0.33 | 0.34 | 0.7228 | 1.03(0.86- 1.24) |  | 0.31 | 0.6866 | 0.92( 0.62- 1.37) |  | 0.31 | 0.7025 | 0.91( 0.56- 1.48) |
| 1 | rs10923445 | | 116320833 | G | 0.40 | 0.40 | 0.9818 | 1.00(0.84- 1.19) |  | 0.43 | 0.5488 | 1.12( 0.77- 1.63) |  | 0.42 | 0.7242 | 1.09( 0.69- 1.71) |
| 3 | rs6771541 | | 27526516 | G | 0.90 | 0.90 | 0.7684 | 1.04(0.79- 1.38) |  | 0.91 | 0.7298 | 1.12( 0.59- 2.10) |  | 0.88 | 0.7632 | 0.90( 0.44- 1.81) |
| 3 | rs9871261 | | 27529889 | C | 0.85 | 0.87 | 0.2849 | 1.14(0.89- 1.46) |  | 0.91 | 0.1009 | 1.68( 0.90- 3.15) |  | 0.87 | 0.596 | 1.20( 0.61- 2.34) |
| 3 | rs9836685 | | 27535796 | A | 0.86 | 0.86 | 0.9009 | 1.02(0.79- 1.30) |  | 0.88 | 0.5846 | 1.17( 0.66- 2.07) |  | 0.88 | 0.5585 | 1.23( 0.61- 2.49) |
| 3 | rs13082711 | | 27537909 | G | 0.05 | 0.05 | 0.6557 | 1.09(0.74- 1.60) |  | 0.05 | 0.8194 | 1.10( 0.48- 2.55) |  | 0.06 | 0.4866 | 1.38( 0.55- 3.47) |
| 3 | rs7635193 | | 27542500 | G | 0.86 | 0.86 | 0.892 | 1.02(0.79- 1.30) |  | 0.89 | 0.4398 | 1.26( 0.70- 2.26) |  | 0.88 | 0.5809 | 1.22( 0.60- 2.46) |
| 3 | rs113102807 | | 27546886 | G | 0.94 | 0.94 | 0.4252 | 1.16(0.80- 1.69) |  | 0.96 | 0.3757 | 1.50( 0.61- 3.72) |  | 0.93 | 0.9635 | 0.98( 0.39- 2.45) |
| 3 | rs820430 | | 27548900 | G | 0.68 | 0.69 | 0.3973 | 1.08(0.90- 1.30) |  | 0.71 | 0.5079 | 1.15( 0.76- 1.72) |  | 0.69 | 0.7848 | 1.07( 0.66- 1.74) |
| 3 | rs2643825 | | 27562613 | G | 0.05 | 0.05 | 0.705 | 1.08(0.73- 1.58) |  | 0.05 | 0.8437 | 1.09( 0.47- 2.51) |  | 0.06 | 0.5047 | 1.37( 0.54- 3.43) |
| 3 | rs34085137 | | 27588131 | A | 0.04 | 0.05 | 0.4365 | 1.17(0.78- 1.76) |  | 0.05 | 0.526 | 1.31( 0.57- 3.04) |  | 0.05 | 0.6142 | 1.30( 0.47- 3.61) |
| 3 | rs17019924 | | 27595912 | A | 0.79 | 0.80 | 0.56 | 1.06(0.86- 1.31) |  | 0.78 | 0.8966 | 0.97( 0.62- 1.52) |  | 0.79 | 0.9082 | 1.03( 0.59- 1.80) |
| 3 | rs9851320 | | 27601508 | A | 0.18 | 0.21 | 0.09917 | 1.19(0.97- 1.47) |  | 0.21 | 0.4949 | 1.17( 0.74- 1.85) |  | 0.18 | 0.955 | 0.98( 0.55- 1.77) |
| 3 | rs9852113 | | 27602032 | A | 0.13 | 0.12 | 0.4681 | 0.91(0.71- 1.17) |  | 0.18 | 0.1541 | 1.42( 0.88- 2.30) |  | 0.18 | 0.2552 | 1.40( 0.78- 2.52) |
| 3 | rs73055818 | | 27608341 | A | 0.04 | 0.04 | 0.6744 | 1.10(0.71- 1.69) |  | 0.05 | 0.4095 | 1.42( 0.61- 3.30) |  | 0.05 | 0.5085 | 1.41( 0.51- 3.92) |
| 3 | rs12639253 | | 27612399 | G | 0.61 | 0.63 | 0.5113 | 1.06(0.89- 1.26) |  | 0.52 | 0.03978 | 0.68( 0.47- 0.98) |  | 0.55 | 0.2769 | 0.78( 0.50- 1.22) |
| 3 | rs9854207 | | 27614316 | C | 0.42 | 0.41 | 0.8127 | 0.98(0.83- 1.16) |  | 0.52 | 0.03071 | 1.50( 1.04- 2.17) |  | 0.45 | 0.5686 | 1.14( 0.73- 1.79) |
| 3 | rs2642925 | | 27615796 | G | 0.09 | 0.12 | 0.05322 | 1.31(1.00- 1.72) |  | 0.12 | 0.2502 | 1.40( 0.79- 2.48) |  | 0.10 | 0.8581 | 1.08( 0.49- 2.37) |
| 3 | rs2643844 | | 27639940 | C | 0.04 | 0.06 | 0.1412 | 1.32(0.91- 1.91) |  | 0.03 | 0.3428 | 0.57( 0.18- 1.83) |  | 0.10 | 0.01437 | 2.48( 1.17- 5.24) |
| 3 | rs2643823 | | 27646114 | A | 0.37 | 0.36 | 0.677 | 0.96(0.81- 1.15) |  | 0.45 | 0.1025 | 1.36( 0.94- 1.98) |  | 0.44 | 0.2611 | 1.30( 0.82- 2.04) |
| 3 | rs55966019 | | 27652791 | A | 0.03 | 0.04 | 0.3036 | 1.28(0.80- 2.03) |  | 0.05 | 0.1383 | 1.88( 0.81- 4.38) |  | 0.03 | 0.8914 | 0.91( 0.22- 3.74) |
| 3 | rs76747642 | | 27659933 | A | 0.94 | 0.94 | 0.9684 | 0.99(0.69- 1.43) |  | 0.91 | 0.1869 | 0.64( 0.33- 1.25) |  | 0.95 | 0.8277 | 1.12( 0.41- 3.09) |
| 3 | rs8179938 | | 27680063 | A | 0.59 | 0.61 | 0.3821 | 1.08(0.91- 1.28) |  | 0.67 | 0.06624 | 1.44( 0.97- 2.14) |  | 0.60 | 0.7837 | 1.07( 0.67- 1.69) |
| 3 | rs3856680 | | 27702442 | A | 0.64 | 0.64 | 0.8082 | 1.02(0.86- 1.22) |  | 0.69 | 0.2532 | 1.26( 0.85- 1.88) |  | 0.63 | 0.8611 | 0.96( 0.60- 1.53) |
| 3 | rs6764447 | | 27721198 | G | 0.68 | 0.72 | 0.08003 | 1.18(0.98- 1.42) |  | 0.73 | 0.267 | 1.27( 0.83- 1.92) |  | 0.72 | 0.5245 | 1.18( 0.71- 1.94) |
| 3 | rs2642935 | | 27726951 | A | 0.96 | 0.97 | 0.2436 | 1.34(0.82- 2.18) |  | 0.97 | 0.4689 | 1.53( 0.48- 4.88) |  | 0.96 | 0.9792 | 1.02( 0.32- 3.26) |
| 3 | rs10936694 | | 171314225 | A | 0.76 | 0.77 | 0.8928 | 1.01(0.83- 1.24) |  | 0.72 | 0.3333 | 0.82( 0.54- 1.23) |  | 0.78 | 0.6965 | 1.11( 0.65- 1.92) |
| 3 | rs2287579 | | 171362785 | A | 0.03 | 0.05 | 0.008368 | 1.71(1.14- 2.55) |  | 0.03 | 0.7674 | 0.84( 0.26- 2.69) |  | 0.01 | 0.3631 | 0.41( 0.06- 2.98) |
| 3 | rs2290480 | | 171404478 | A | 0.12 | 0.13 | 0.4112 | 1.11(0.86- 1.43) |  | 0.18 | 0.03789 | 1.66( 1.02- 2.70) |  | 0.22 | 0.006861 | 2.10( 1.21- 3.62) |
| 3 | rs7649974 | | 171409603 | G | 0.31 | 0.33 | 0.3353 | 1.09(0.91- 1.31) |  | 0.42 | 0.008847 | 1.64( 1.13- 2.39) |  | 0.41 | 0.05335 | 1.56( 0.99- 2.47) |
| 3 | rs6773529 | | 171449100 | G | 0.57 | 0.56 | 0.6168 | 0.96(0.81- 1.14) |  | 0.59 | 0.6753 | 1.09( 0.74- 1.59) |  | 0.66 | 0.1232 | 1.46( 0.90- 2.35) |
| 3 | rs9860368 | | 171489402 | C | 0.12 | 0.13 | 0.5657 | 1.08(0.84- 1.39) |  | 0.18 | 0.05941 | 1.59( 0.98- 2.57) |  | 0.24 | 0.001369 | 2.31( 1.36- 3.92) |
| 3 | rs13098743 | | 171495180 | A | 0.04 | 0.04 | 0.5431 | 1.14(0.75- 1.75) |  | 0.04 | 0.7299 | 1.17( 0.47- 2.93) |  | 0.03 | 0.5998 | 0.69( 0.17- 2.83) |
| 3 | rs6767600 | | 171506951 | C | 0.65 | 0.66 | 0.5765 | 1.05(0.88- 1.26) |  | 0.67 | 0.5489 | 1.13( 0.76- 1.67) |  | 0.67 | 0.6971 | 1.10( 0.68- 1.77) |
| 3 | rs12632766 | | 171511355 | G | 0.71 | 0.74 | 0.2421 | 1.12(0.93- 1.36) |  | 0.77 | 0.2061 | 1.33( 0.86- 2.05) |  | 0.83 | 0.02017 | 2.01( 1.10- 3.66) |
| 3 | rs13321742 | | 171519793 | G | 0.69 | 0.68 | 0.8116 | 0.98(0.82- 1.17) |  | 0.69 | 0.9696 | 1.01( 0.68- 1.50) |  | 0.79 | 0.04356 | 1.76( 1.01- 3.06) |
| 3 | rs4894773 | | 171529101 | A | 0.64 | 0.64 | 0.8686 | 1.02(0.85- 1.21) |  | 0.66 | 0.6215 | 1.10( 0.75- 1.63) |  | 0.65 | 0.8213 | 1.06( 0.66- 1.69) |
| 10 | rs138127971 | | 43191350 | G | 0.03 | 0.05 | 0.1051 | 1.40(0.93- 2.12) |  | 0.03 | 0.9602 | 1.03( 0.37- 2.83) |  | 0.05 | 0.3666 | 1.60( 0.57- 4.45) |
| 10 | rs2484304 | | 43193161 | G | 0.96 | 0.98 | 0.06284 | 1.66(0.97- 2.86) |  | 0.97 | 0.8387 | 1.11( 0.40- 3.06) |  | 0.96 | 0.9891 | 0.99( 0.31- 3.19) |
| 10 | rs7898080 | | 43197926 | A | 0.92 | 0.95 | 0.002092 | 1.81(1.23- 2.64) |  | 0.97 | 0.05784 | 2.56( 0.94- 6.98) |  | 0.94 | 0.537 | 1.33( 0.53- 3.33) |
| 10 | rs12265862 | | 43211650 | A | 0.19 | 0.27 | 6.53E-06 | 1.56(1.29- 1.90) |  | 0.25 | 0.1016 | 1.43( 0.93- 2.19) |  | 0.27 | 0.07547 | 1.58( 0.95- 2.62) |
| 10 | rs11239753 | | 43224684 | A | 0.03 | 0.04 | 0.03165 | 1.60(1.04- 2.46) |  | 0.09 | 0.0002548 | 3.32( 1.68- 6.55) |  | 0.09 | 0.00125 | 3.47( 1.55- 7.75) |
| 10 | rs10508868 | | 43224701 | G | 0.03 | 0.05 | 0.07047 | 1.45(0.97- 2.18) |  | 0.09 | 0.0004791 | 3.01( 1.57- 5.76) |  | 0.09 | 0.007621 | 2.83( 1.27- 6.30) |
| 10 | rs17153309 | | 43225728 | A | 0.19 | 0.28 | 3.62E-07 | 1.64(1.35- 1.99) |  | 0.27 | 0.04989 | 1.52( 1.00- 2.31) |  | 0.26 | 0.1671 | 1.44( 0.86- 2.40) |
| 10 | rs1414027 | | 43240428 | A | 0.68 | 0.75 | 0.000312 | 1.42(1.17- 1.73) |  | 0.75 | 0.1108 | 1.41( 0.92- 2.16) |  | 0.74 | 0.2326 | 1.37( 0.82- 2.28) |
| 10 | rs624804 | | 43253587 | A | 0.37 | 0.44 | 0.001016 | 1.33(1.12- 1.58) |  | 0.47 | 0.03206 | 1.50( 1.03- 2.17) |  | 0.36 | 0.8743 | 0.96( 0.60- 1.54) |

A1, reference allele, F_U, frequency of A1 allele in controls. F_A, frequency of A1 allele in cases.

Supplementary Table S4. Allele frequencies of four SNPS in previous GWAS study and current study.

| SNP | Alleles ^a^ | Genes | Minor allele frequency | | OR (95% CI) | *P* value | Study |
| --- | --- | --- | --- | --- | --- | --- | --- |
|  |  |  | Cases | Controls |  |  |  |
| rs9428225 | A/G | *CASQ2* | 0.448 | 0.434 | 1.06 [0.91-1.24] | 0.445 | Present study |
|  |  |  | 0.472 | 0.356 | 1.62 [1.34-1.96] | 6.6×10^-7^ | Tang et al. ^b^ |
| rs9851320 | A/C | *SLC4A7*-*EOMES* | 0.213 | 0.181 | 1.22 [1.01-1.47] | 0.039 | Present study |
|  |  |  | 0.248 | 0.164 | 1.84 [1.44-2.34] | 9.2×10^-7^ | Tang et al. |
| rs12632766 | A/G | *PLD1* | 0.251 | 0.286 | 0.83 [0.70-0.99] | 0.04 | Present study |
|  |  |  | 0.196 | 0.293 | 0.57 [0.45-0.71] | 7.4×10^-7^ | Tang et al. |
| rs1414027 | T/A | *LINC01518*-*LOC283028* | 0.247 | 0.320 | 0.70 [0.59-0.83] | 4.6×10^-5^ | Present study |
|  |  |  | 0.210 | 0.325 | 0.55 [0.44-0.69] | 1.4×10^-7^ | Tang et al. |

a. The allele above the slash is the minor allele.

b. Four associated SNPs identified in the previous GWAS by Tang et al. in 2018.
